# Supplementary material for: Causal Relationship between Aging and Anorexia Nervosa: A White-Matter-Microstructure-Mediated Mendelian Randomization Analysis
Source: Biomedicines. 2024 Aug 16;12(8):1874. doi: 10.3390/biomedicines12081874 (PMC11351342; doi:10.3390/biomedicines12081874)
Supplement: Supplementary file 1 [file biomedicines-12-01874-s001.zip › Supplementary Figure/Figure S4/Singe SNP plot of MR analysis for TL to ubm-b-2006.pdf]

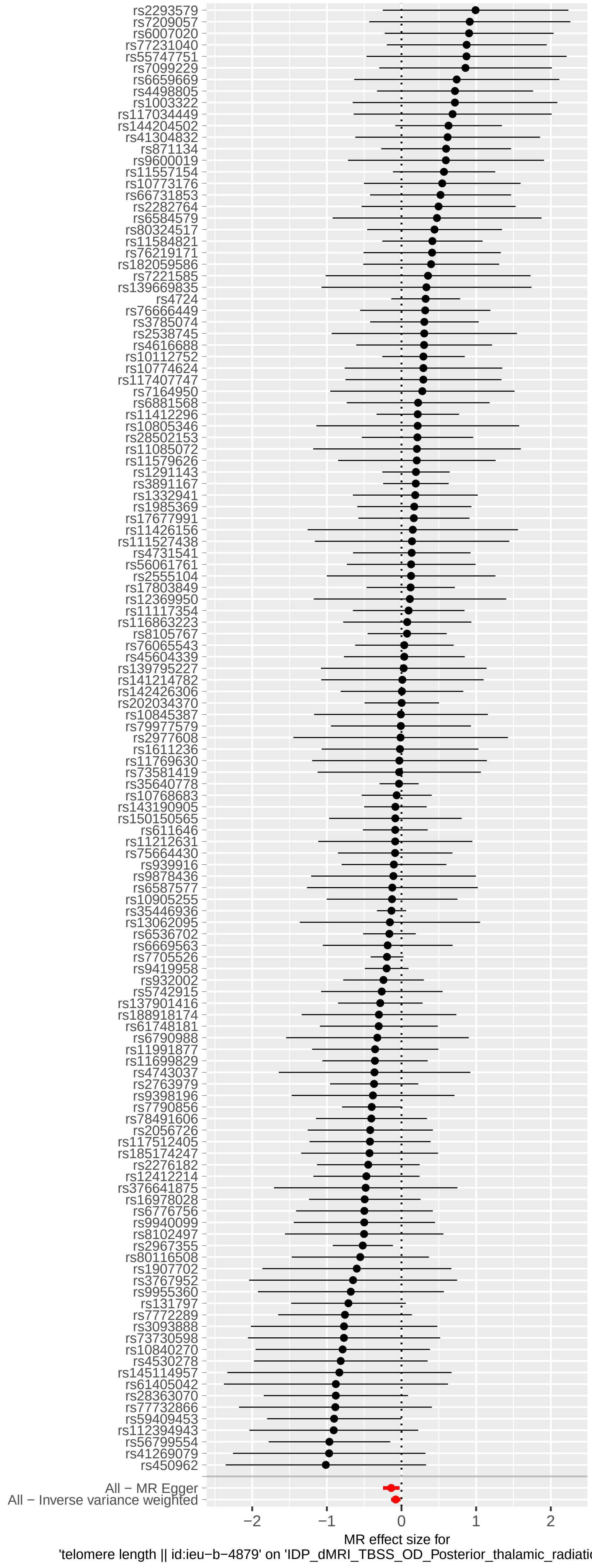
rs2293579
rs7209057
rs6007020
rs77231040
rs55747751
rs7099229
rs6659669
rs4498805
rs1003322
rs117034449
rs144204502
rs41304832
rs871134
rs9600019
rs11557154
rs10773176
rs66731853
rs2282764
rs6584579
rs80324517
rs11584821
rs76219171
rs182059586
rs7221585
rs139669835
rs4724
rs76666449
rs3785074
rs2538745
rs4616688
rs10112752
rs10774624
rs117407747
rs7164950
rs6881568
rs11412296
rs10805346
rs28502153
rs11085072
rs11579626
rs1291143
rs3891167
rs1332941
rs1985369
rs17677991
rs11426156
rs111527438
rs4731541
rs56061761
rs2555104
rs17803849
rs12369950
rs11117354
rs116863223
rs8105767
rs76065543
rs45604339
rs139795227
rs141214782
rs142426306
rs202034370
rs10845387
rs79977579
rs2977608
rs1611236
rs11769630
rs73581419
rs35640778
rs10768683
rs143190905
rs150150565
rs611646
rs11212631
rs75664430
rs939916
rs9878436
rs6587577
rs10905255
rs35446936
rs13062095
rs6536702
rs6669563
rs7705526
rs9419958
rs932002
rs5742915
rs137901416
rs188918174
rs61748181
rs6790988
rs11991877
rs11699829
rs4743037
rs2763979
rs9398196
rs7790856
rs78491606
rs2056726
rs117512405
rs185174247
rs2276182
rs12412214
rs376641875
rs16978028
rs6776756
rs9940099
rs8102497
rs2967355
rs80116508
rs1907702
rs3767952
rs9955360
rs131797
rs7772289
rs3093888
rs73730598
rs10840270
rs4530278
rs145114957
rs61405042
rs28363070
rs77732866
rs59409453
rs112394943
rs56799554
rs41269079
rs450962
All − MR Egger
All − Inverse variance weighted
−2
−1
0
1
2
MR effect size for
'telomere length || id:ieu−b−4879' on 'IDP_dMRI_TBSS_OD_Posterior_thalamic_radiatio
